# A deep learning model incorporating part of speech and self-matching attention for named entity recognition of Chinese electronic medical records

**DOI:** 10.1186/s12911-019-0762-7

**Published:** 2019-04-09

**Authors:** Xiaoling Cai, Shoubin Dong, Jinlong Hu

**Affiliations:** Communication & Computer Network Lab of Guangdong, School of Computer Science and Engineering, South China University of Technology, Guangzhou, China

**Keywords:** Part of speech, Chinese electronic medical records, Named entity recognition

## Abstract

**Background:**

The Named Entity Recognition (NER) task as a key step in the extraction of health information, has encountered many challenges in Chinese Electronic Medical Records (EMRs). Firstly, the casual use of Chinese abbreviations and doctors’ personal style may result in multiple expressions of the same entity, and we lack a common Chinese medical dictionary to perform accurate entity extraction. Secondly, the electronic medical record contains entities from a variety of categories of entities, and the length of those entities in different categories varies greatly, which increases the difficult in the extraction for the Chinese NER. Therefore, the entity boundary detection becomes the key to perform accurate entity extraction of Chinese EMRs, and we need to develop a model that supports multiple length entity recognition without relying on any medical dictionary.

**Methods:**

In this study, we incorporate part-of-speech (POS) information into the deep learning model to improve the accuracy of Chinese entity boundary detection. In order to avoid the wrongly POS tagging of long entities, we proposed a method called reduced POS tagging that reserves the tags of general words but not of the seemingly medical entities. The model proposed in this paper, named SM-LSTM-CRF, consists of three layers: self-matching attention layer – calculating the relevance of each character to the entire sentence; LSTM (Long Short-Term Memory) layer – capturing the context feature of each character; CRF (Conditional Random Field) layer – labeling characters based on their features and transfer rules.

**Results:**

The experimental results at a Chinese EMRs dataset show that the F1 value of SM-LSTM-CRF is increased by 2.59% compared to that of the LSTM-CRF. After adding POS feature in the model, we get an improvement of about 7.74% at F1. The reduced POS tagging reduces the false tagging on long entities, thus increases the F1 value by 2.42% and achieves an F1 score of 80.07%.

**Conclusions:**

The POS feature marked by the reduced POS tagging together with self-matching attention mechanism puts a stranglehold on entity boundaries and has a good performance in the recognition of clinical entities.

## Background

Since the implementation of the “Basic Norms of Electronic Medical Records” in China, the number of Chinese Electronic Medical Records (EMRs) has increased dramatically, and the Named Entities Recognition (NER) on EMRs has received constant research attention over recent years. NER automatically identifies entities related to the patient and produces structured data to help constructing the knowledge graph in medical domain [1–4].

Electronic medical records are usually written by doctors, sometimes using Chinese abbreviations, resulting in multiple expressions of the same entity. For example, “贝伐”, “贝伐珠单抗” and “贝伐单抗” all refer to the same drug “bevacizumab”. And compared with English clinical entities, the composition of Chinese clinical entities is more complicated with mixed Chinese characters, letters, numbers and punctuations, adding the difficulties in identifying the entities. More importantly, the Chinese entities are not only generally longer than that English entities, but are varied in length greatly among different categories. For example, the “body” entities are short and may only be of 1–2 characters, such as “头” (head), while the “operation” entities are longer, sometimes as long as more than 20 characters. In fact, the above problem can be classified as the boundary extraction problem of Chinese entities. The Chinese named entity recognition task can be divided into two key steps, entity category extraction and entity boundary extraction. This paper focuses on the problem of entity boundary extraction with different entity lengths, and proposes a deep learning model that combines part-of-speech information and self-matching attention mechanism for name entity recognition of Chinese electronic medical records.

In the traditional NER method based on machine learning, part-of-speech information is considered as an important feature of entity recognition [5–9]. K. Liu et al. [9] extracted characters and part-of-speech features from Chinese clinical electronic medical records and studied their effects on the NER task. They presumed that because disease entities are mostly composed of several consecutive nouns and treatment entities often occur after verbs, the POS feature can be helpful in improving the recognition of clinical entities. W. Li et al. [10] added the part-of-speech information into the deep belief network (DBN) and found that the improved DBN method is not only superior to the original DBN method, but also exceeds CRF method at the F value. The authors thus concluded that the model can capture semantic information after added with part-of-speech information.

However, the POS feature is only rarely incorporated in the neural network models. One of the reasons is that the current part-of-speech tagging of electronic medical records is not accurate enough, and there are many errors in the tagging results. The error propagation leads to poor entity recognition. For example, “部分小肠切除术” (partial small bowel resection) is a Chinese “operation” entity that can be marked as two parts “部分小肠_n 切除术_v” by POS tagging tools, and the system may only pick out “部分小肠” (partial small intestine) as an “operation” entity. The above-mentioned papers [10] solved this problem by constructing a medical dictionary, which took a lot of efforts. In this paper, we propose a reduced part-of-speech tagging method, which only labels the general vocabularies rather than the clinical entities, to enhance the labeling accuracy of long entities.

In order to extract features automatically, neural networks are applied to NER task. Most of NER deep learning models are based on LSTM-CRF and its variants [11–14]. The recent research on neural networks proposed the use of “attention mechanisms” that rely on weighted representations in multiple time steps along a sequence, rather than relying solely on a single representation of the entire sequence [15]. The attention mechanism allows the model to better focus on the relevant parts of the input and has proven to greatly improve the performance of the loop network in various natural language processing tasks [16–19]. The study [20] evaluated the self-attention mechanism empirically on the CoNLL-2003 dataset and on synthetic datasets with particularly long-term relationships between words and showed that the approach achieves a much better performance in long-term interdependent text sequences. Z. Liu et al. [21] proposed an attention-based CNN-LSTM-CRF model for extracting Chinese clinical entities, which utilizes an attention layer to select relevant words.

In this study, we proposed a SM-LSTM-CRF model design for NER task, which is based on self-matching attention mechanism. We use part-of-speech features to introduce entity part-of-speech information and sentence semantic information. And the self-attention mechanism is added to our model to solve long-dependency problems and improve the accuracy of entity boundaries recognized. Particularly, we propose a reduced part-of-speech tagging method to reduce the markup errors of long entities.

## Method

### Data preprocessing

As name entities become the main source of out of vocabulary (OOV) in segmentation task, the NER methods should have the ability to overcome the potential issue of incorrectly segmented entity boundaries. A few researches showed that character-based methods outperform word-based methods for Chinese NER [7, 9]. But there is still a problem that the words are generally used as the smallest unit to express semantics in Chinese language. Though the character-based model may avoid segmentation error, it loses part of semantic as well. On the other hand, character sequence is always longer than word sequence, increasing the difficulties with entity boundary extraction. To tackle those problems, we added the part-of-speech features to the model and concatenated it with characters as the input of model.

Part-of-speech (POS) refers to the characteristics of words as the basis for classifying words, including nouns, verbs, adjectives, modal particles etc. Prior work based on rules or machine learning has shown that POS can dramatically improve NER annotation, and suggested the possibility of increasing accuracy through adding POS to the deep neural network. We add POS feature into the deep learning model for the following considerations:

1. The POS information implies potential word segmentation, which indirectly adds semantics to the model. Although the entity is divided into multiple characters in preprocessing, those characters have the same POS tag as that entity, which making the boundary easier to determine.
2. Most of name entities are OOV that cannot be correctly labeled during POS tagging. While the common words can be tagged exactly, to the benefit of distinguishing the edges between the medical entities and the ordinary words.
3. In different word contexts, the same character can be marked as different POS tags, which suggest the possibility of increasing the accuracy of entity category recognized.

When performing POS tagging on electronic medical records by tools, the long entities may be tagged as multiple parts, which may result in recognition errors for the long clinical entity. For example, the entity “部分小肠切除术”(partial resection of small bowel) is marked as two parts: “部分小肠_n 切除术_v”, and the system may pick out “部分小肠” (partial small intestine) as an “operation” entity. It is traditional that medical dictionaries are added to help identify the medical entities, but because of the complexity of building medical dictionaries, we propose a reduced part-of-speech tagging method, which construct a general dictionary instead of a medical dictionary.

We first utilize the Jieba tool [22] to perform word segmentation on the training data, gathering the non-entity word segments to get the general vocabulary dictionary. After tagging the POS of the training and the test data, reserve the POS tags of the word segments that are included in the dictionary and classify those not as character sequences which are then given POS of “s”.

**Fig. 1:**
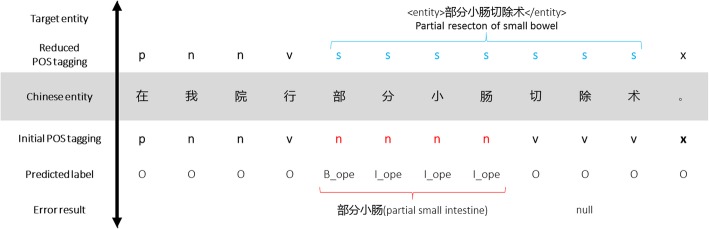
Example for reduced POS tagging

As shown in the Fig. 1 (“O” represents ordinary words, “B_ope” the beginning characters of the “operation” entities and “I_ope” the middle characters of the “operation” entities.), after using the reduced POS tagging, the boundaries of the clinical entity become more distinguishable, which is indeed beneficial to the boundary extraction of the entities. Meanwhile, the tags of the context are kept, making it possible to improve the accuracy of entity category recognized by learning the POS features of the context. Lastly, the common dictionary costs far less to build than the medical dictionary, and the method excluding the need for medical expert tagging can be conveniently applied to another dataset.

**Fig. 2:**
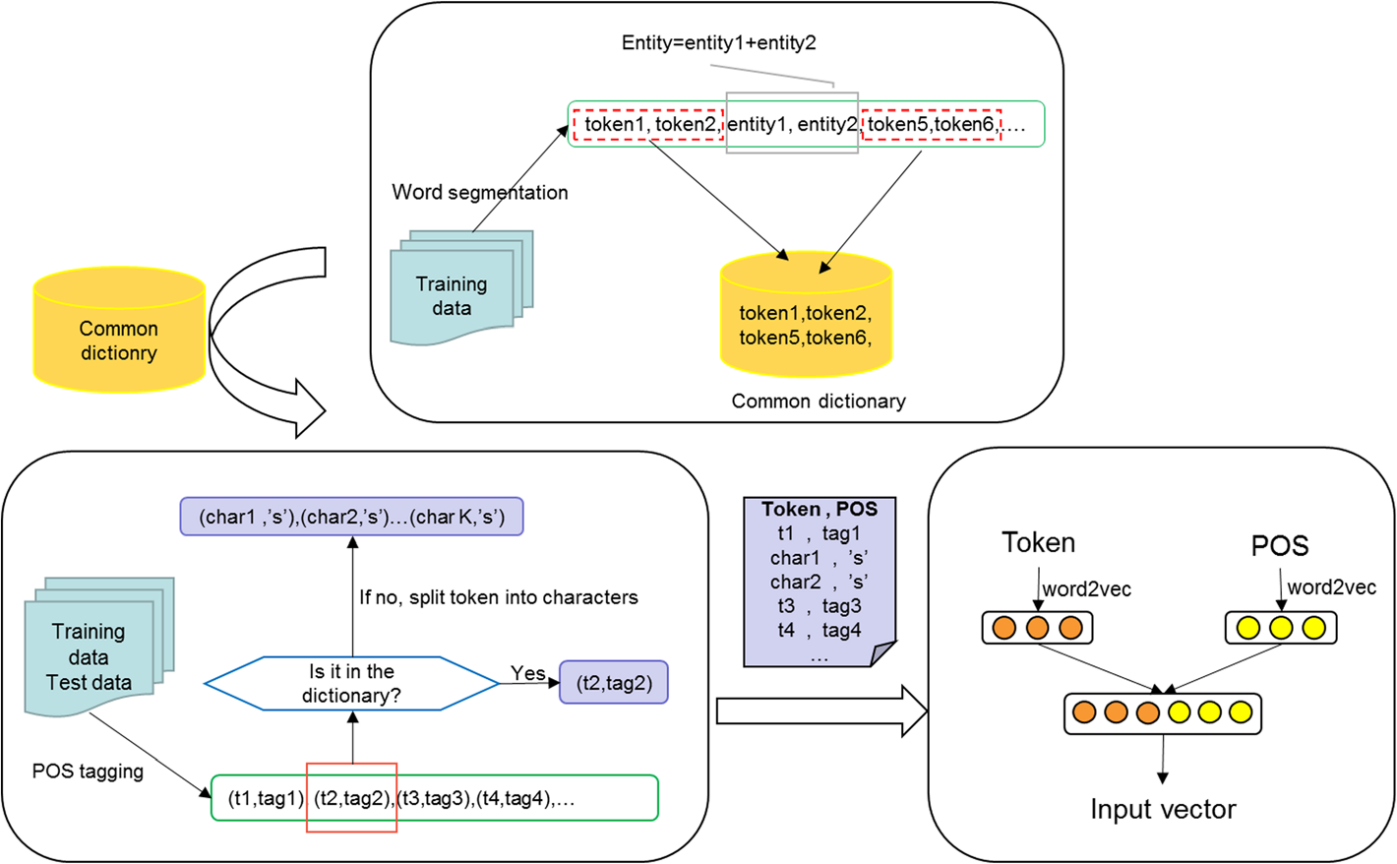
The data preprocessing flowchart

Formally, denote the character vectors as $X={\left\{{x}_t\right\}}_{t=1}^m,{x}_t\in {R}^l$ and POS vectors as $P={\left\{{p}_t\right\}}_{t=1}^m,{p}_t\in {R}^l$, where *l* is the vector dimension and *m* is the length of the sentence. And the input of model as *V* = {*X*; *P*}. The data preprocessing process is shown in the Fig. 2.

### SM-LSTM-CRF model

**Fig. 3:**
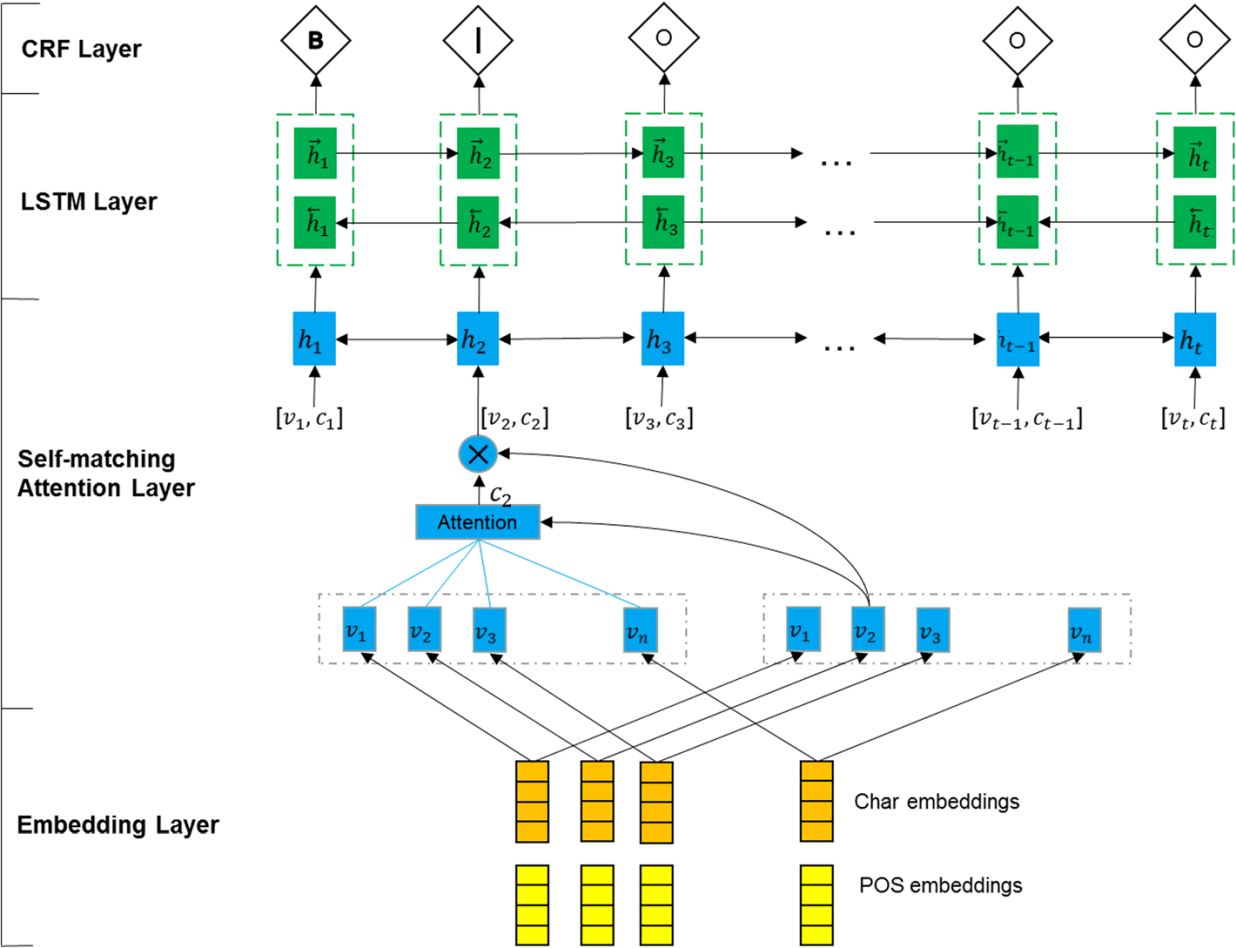
The structure of SM-LSTM-CRF model

The model proposed in this paper called SM-LSTM-CRF, which is the LSTM-CRF model based on the self-matching attention mechanism. The model consists of three layers: self-matching attention layer – calculating the relevance of each character to the entire sentence; LSTM layer – capturing the context feature of each character; CRF layer – labeling characters based on their features and transfer rules. The structure is shown in Fig. 3.

In Chinese EMRs, there are some types of entities having a longer length, which need to consider a farther sequence dependency. Recently attention mechanisms are proposed in sequence labeling to overcome this problem by allowing directly the use of information from across all time steps. Self-matching attention calculates the weight dependent on the input sequence and gets the attention of each characters across the sentence. Self-matching attention performs parameter calculations through a bidirectional LSTMs network, and the input of each time step is modified to be [*v*_*t*_, *c*_*t*_], where *v*_*t*_ is the input vector at time step *t* and *c*_*t*_ is the attention vector of *v*_*t*_ related to the whole sentence.

1 $$ {h}_t= LSTM\left({h}_{t-1},\left[{v}_t,{c}_t\right]\right) $$

Where *c*_*t*_ = *att*(*V*, *v*_*t*_) is an attention-pooling vector of the whole sentence.

2 $$ {s}_j^t={W}^T\tanh \left({W}_v{v}_j+{W}_{\widehat{v}}{v}_t\right) $$

3 $$ {a}_i^t=\exp \left({s}_i^t\right)/\sum \limits_{j=1}^m\exp \left({s}_j^t\right) $$

4 $$ {c}_t=\sum \limits_{i=1}^m{a}_i^t{v}_i $$

We further build a similar self-matching in the reverse direction, to obtain a representation that encodes the contexts from both directions for each token.

5 $$ \overrightarrow{h_t}=\overrightarrow{LSTM}\left(\overrightarrow{h_{t-1}},\left[{v}_t,{c}_t\right]\right) $$

6 $$ \overleftarrow{h_t}=\overleftarrow{\mathrm{LSTM}}\left(\overrightarrow{h_{t-1}},\left[{v}_t,{c}_t\right]\right) $$

7 $$ H={\left\{\overrightarrow{h_t};\overleftarrow{h_t}\right\}}_{t=1}^m $$

The resulting matrices $H={\left\{{h}_t\right\}}_{t=1}^m,{h}_t\in {R}^{2k}$ are hidden representations of the sentence, where *m* is the length of input and *k* is hidden unit of LSTM.

The recoded matrix is inputted into the bidirectional LSTMs to learn contextual feature. Compared to a simple RNN, the LSTM is better in learning context-dependent feature around the input vector. We use a standard directional LSTMs to process the recode embedding as shown below:

8 $$ L= BiLSTM(H) $$

The resulting matrix: $L={\left\{{l}_t\right\}}_{t=1}^m,{l}_t\in {R}^{2k}$, where *k* is hidden unit of LSTM.

A standard CRF layer is used on top of model and the output of LSTM layer is converted to an input by a linear function.

9 $$ P=L{W}_p+{b}_p $$

Where *W*_*p*_ ∈ *R*^2*k* × *n*^, *b*_*p*_ ∈ *R*^*n*^ are parameters to be learned, *n* is the number of tag types. The input matrices of CRF is *P*, where *P*_*i*, *j*_ represents the non-normalized probability that the characters are mapped to the name entity labels. The transition probability matrix *A* is learned in CRF and *A*_*i*, *j*_ represents the transition probability when a label transfers to another label. For an input *V*, the probability of outputting the optimal tag sequence y can be defined as:

10 $$ p\left(y|V\right)=\frac{e^{s\left(V,y\right)}}{\sum_{\overset{\sim }{y}\in {Y}_V}{e}^{s\left(V,\overset{\sim }{y}\right)}} $$

11 $$ s\left(V,y\right)=\sum \limits_{i=0}^m{A}_{y_i,{y}_{i+1}}+\sum \limits_{i=1}^m{P}_{i,{y}_i} $$

During the model training process, the loss function is defined by:

12 $$ -\mathit{\log}\left(p\left(y|V\right)\right)=-\mathit{\log}\left(\frac{e^{s\left(V,y\right)}}{\sum_{\overset{\sim }{y}\in {Y}_V}{e}^{s\left(V,\overset{\sim }{y}\right)}}\right) $$

By minimizing the loss function, the probability of the optimal labeling sequence is increased, and the label transition probability matrix is obtained during the training of the model parameters.

## Results

### Dataset

The data set includes 1000 admission records for patients, which are adopted from the Chinese EMR named entity recognition task in China Conference on Knowledge Graph and Semantic Computing in 2018 (http://www.ccks2018.cn/).

Table 1 shows the number of entities and the average number of characters per entity both in training datasets and testing datasets. In the experiment, 600 of the records are used as training data, and the remaining are test data. The entities in patient admission records are mainly divided into five categories:

1. Anatomical Part: the functional structural unit made up of a variety of organizations, such as “腹部” (abdomen).
2. Symptom Description: refers to the patient’s own experience and feeling of abnormal physiological function after the patient is sick, and needs to be combined with the anatomical part, such as “不适” (discomfort).
3. Independent Symptoms: refers to the patient’s own experience and feeling of the body’s physiological function after illness, can be independently output, such as “眩晕” (dizziness).
4. Drug: a kind of chemical substances used for curing, preventing or promoting health.
5. Operation: refers to excision, suture and other treatments operated on the patient by a surgeon.

**Table 1:** The number of entities and the average number of characters per entity

	Anatomical Part	Symptom Description	Independent Symptoms	Drug	Operation
Train Set	7838/2.5	2066/1.5	3055/2.5	1005/3.4	1116/7.9
Test Set	6339/2.7	918/1.6	1327/2.8	813/3.4	735/7.3

### Implementation and parameters

Our neural networks are implemented on Python 2.7.13 and Tensorflow 1.8.0 library, and the POS tagging are performed by Jieba 0.39. We cut the sentences to segments by the Chinese punctuation marks such as comma and period. Table 2 shows the adopted hyper-parameters about our model. A model is designed to stop training, when the CRF loss of the two epoch of training did not differ by more than 0.001.

**Table 2:** Hyper-parameters

Parameter	Value
Character embedding size	50
POS embedding size	50
Initial learning rate	0.001
Batch size	32
Maximum training epochs	20
Size of LSTM hidden units	200
Optimizer	Adam

### Evaluation metrics

The evaluation metrics, namely, Precision (P), Recall (R) and F1-measure (F1) are used to evaluate the performance of the NER methods, and defined as follows:

$$ \mathrm{P}=\frac{TP}{TP+ FP} $$

$$ \mathrm{R}=\frac{TP}{TP+ FN} $$

$$ \mathrm{F}1=\frac{2\times P\times R}{P+R} $$

An entity is annotated as correct when its category and boundary are fully labeled correctly. TP is the count of entities labels presenting the same labels as gold standard labels, FP is the count of recognized entities marked incorrectly in the results and FN is the count of the gold standard entities not present in the results of indicator.

### Experimental result

#### The effect of self-matching attention mechanism

As listed in Table 3, we firstly examine the performance of Self-matching attention mechanism by comparing SM-LSTM-CRF with LSTM-CRF, both with an input of character vectors instead of word vectors.

**Table 3:** Performance of Self-matching attention mechanism

	P	R	F
LSTM-CRF	0.6568	0.6904	0.6732
SM-LSTM-CRF	0.6858	0.6991	0.6991

The above table shows that SM-LSTM-CRF achieves a better performance compared to the LSTM-CRF with F1 improved 2.59%. The simple LSTM-CRF model copes quite well with easier instances and performs well in short entities. But as shown in Table 1, there are some longer entities such as “operation” entities which have an average length of 8 characters. However, the self-matching attention mechanism can detect features of input space regardless of position, which exhibits good performance for the more difficult instances and improves the prediction of long entity boundaries.

#### The effect of part-of-speech (POS)

In order to examine the impact of POS on NER, we add POS feature to the LSTM-CRF and SM-LSTM-CRF. In this section, we compare two different input processing, one using only the character vectors as input, the other the concatenation of the character vectors and the POS vectors. The POS is tagged by the Jieba tool.

As shown in Table 4, the performance of the LSTM-CRF and the SM-LSTM-CRF proposed in this paper both improve significantly after adding POS feature. The POS feature introduces boundary and semantic information to offset the weakness of character-based input. The LSTM-CRF model has outperformed by 8.14% and the SM-LSTM-CRF increases F1 by 7.74%, which implies that POS is beneficial to enhance the performance of the deep learning model applied for NER.

**Table 4:** Performance after adding POS information

	LSTM-CRF		SM-LSTM-CRF	
	Character	Character + POS	Character	Character + POS
P	0.6568	0.7606	0.6858	0.7819
R	0.6904	0.7487	0.6991	0.7713
F	0.6732	0.7546	0.6991	0.7765

#### Comparison the impact of different part-of-speech tagging methods

In this section, we compare the effects of two part-of-speech tagging methods on the recognition of entities. One is tagged by the Jieba tool and the other is the reduced POS tagging proposed in this paper. To verify the generality of this preprocessing method, we performed experiments on LSTM-CRF and SM-LSTM-CRF.

As shown in Table 5, the reduced POS tagging method proposed in this paper has a good performance on both two models with the F1 increased respectively by 3.48 and 2.42%. In other words, it improves the extraction accuracy rate of entity boundaries through the enhanced distinguishability of the entities.

**Table 5:** Comparison of the different POS tagging methods

	LSTM-CRF		SM-LSTM-CRF	
	Initial POS tagging	Reduced POS tagging	Initial POS tagging	Reduced POS tagging
P	0.7606	0.7959	0.7819	0.8054
R	0.7487	0.7829	0.7713	0.7961
F	0.7546	0.7894	0.7765	0.8007

#### Comparison with other algorithms

In this section, the work of this paper is compared with other algorithms. The results are shown in Table 6.

**Table 6:** Performance comparison of different algorithms

	P	R	F1
N-gram-Based RNN-CRF [23]	0.5254	0.4056	0.4578
Character-Based LSTM-CRF [11]	0.6568	0.6904	0.6732
Attention-Based CNN-LSTM-CRF [21]	0.7224	0.7248	0.7236
SM-LSTM-CRF	0.8054	0.7961	0.8007

The N-gram-Based RNN-CRF model has a complex processing for the input representation. The author concatenated the current character with its bi-gram, tri-gram representation, which leads to a larger dimension for input vectors. While the idea of the paper is worth learning, it may not be applicable to the current data set. The character-based LSTM-CRF has an F1 value of about 67.32%. In the attention-based CNN-LSTM-CRF, the author used the Xinhua dictionary to extract the radicals of Chinese characters, which were then used to calculate the similarities between characters through the CNN layer. Compared with other algorithms, the SM-LSTM-CRF has a better performance on the current task. What’s more, the processing in this paper is simple without the need for any medical dictionary.

## Discussion

**Fig. 4:**
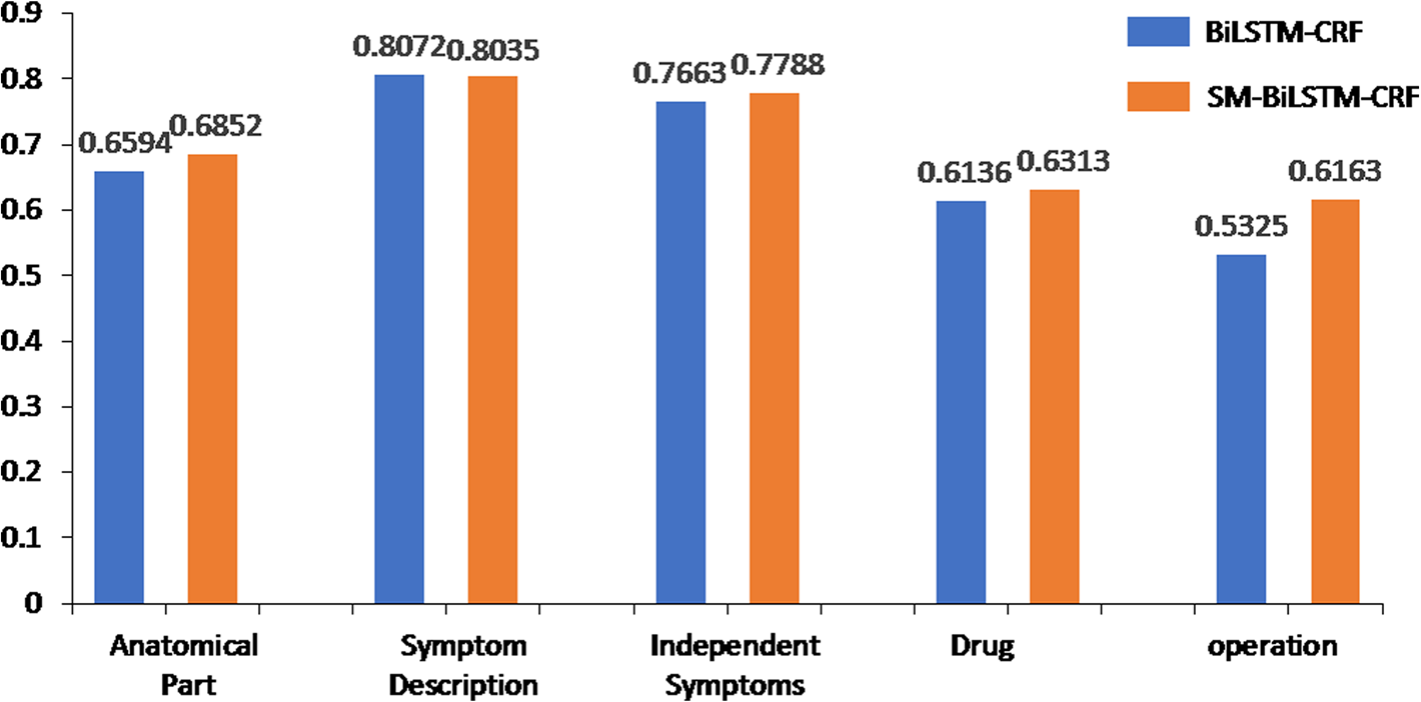
Performance comparison of different models on the five types of entities

The experimental results shown in Table 3 demonstrate that the self-matching attention mechanism performs well in entity recognition. We analyze its performance on the five types of entities shown in Fig. 4. It can be seen that SM-LSTM-CRF model performs better in the “operation” categories with F1 increased by 7.6%. There is a context model around the “operation” entities. Take “行 < entity>直肠癌根治术</entity>”(perform <entity>radical resection of rectal cancer</entity>) as an example: the Chinese character “行” (perform) always appears before “operation” entities, and often incorrectly labeled as part of a “operation” entity or labeled as a “operation” entity itself by the LSTM-CRF model. However, this error can be effectively decreased after adding the self-matching attention mechanism. The self-matching attention mechanism enhances the correlation between the characters in the entity by calculating the relevancy between characters, thus improves the recognition accuracy rate of the entity boundaries with less errors made.

We statistically analyzes the distribution of entities in the incorrect results of the two models, and get the number of entities with “boundary correct, category error”, “boundary error, category correct” and “boundary error, category error” as shown in Table 7. It can be seen that most of the errors are caused by inaccurate boundary while the category errors only make up a small proportion. Therefore, for the NER model with characters as input, we can improve the performance by increasing the accuracy of entity boundary. Compared with the LSTM-CRF, SM-LSTM-CRF model reduces the number of entities with wrong boundary and improves the accuracy of entity labeling. We emphasize that the self-matching attention mechanism improves the accuracy of the entity boundary recognized, especially for the entities with a long length.

**Table 7:** The distribution of entities in the incorrect results

	Correct boundary, Wrong category	Wrong boundary, Correct category	Wrong boundary, Wrong category
LSTM-CRF	67	1863	987
SM-LSTM-CRF	60	1357	446

**Fig. 5:**
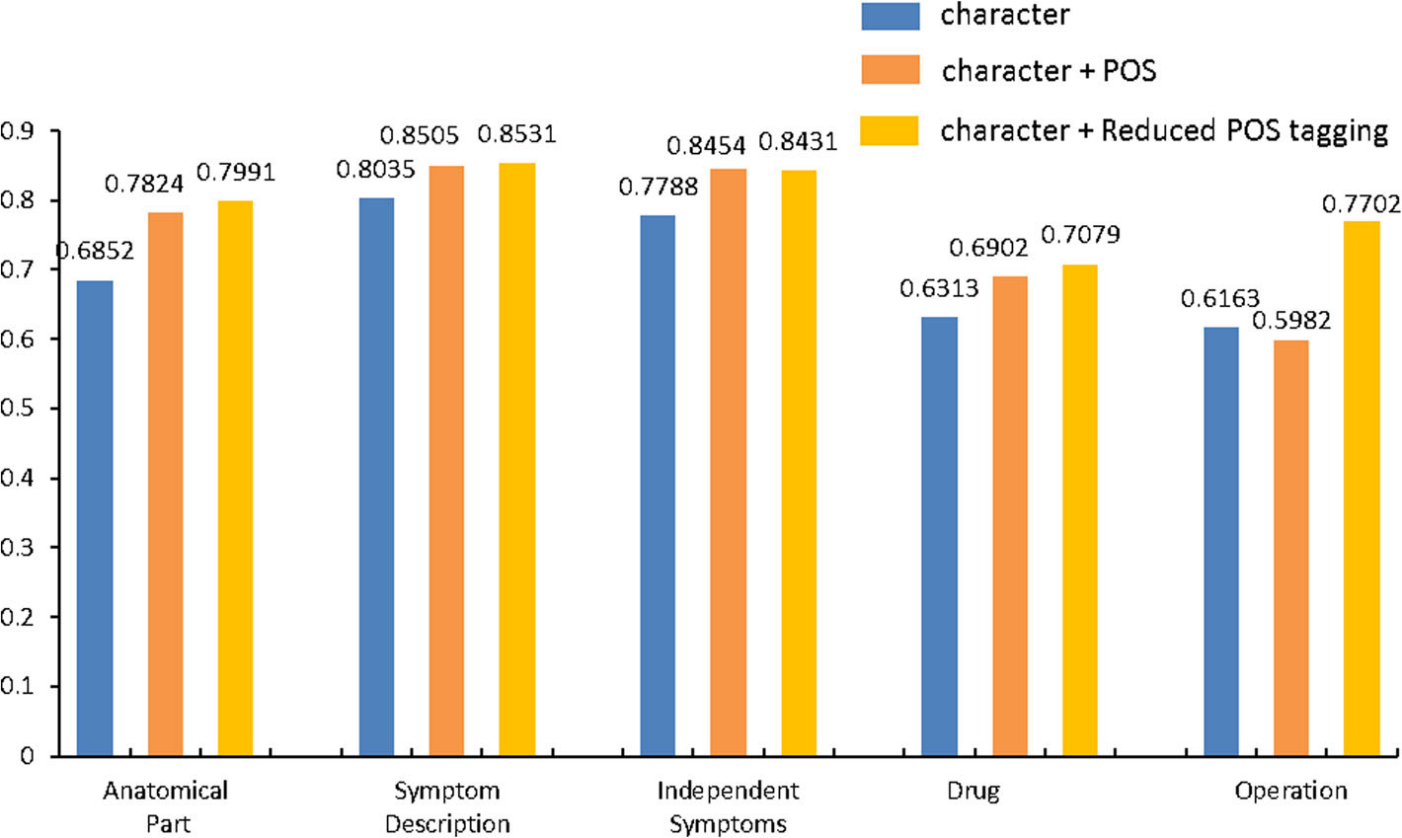
Performance comparison of different input methods on the five types of entities

In addition, we compare the performance of three types of input used for SM-LSTM-CRF on the five categories of entities. As shown in Fig. 5, after adding the POS, the model performs well on the “Anatomical Part”, “Symptom Description”, “Independent Symptoms” and “Drug” entities, but has a reduce of F1 value in “Operation” entity. As we have shown in Table 1, the entities of “Operation” have a longer character sequence and the characters are marked with different POS tags, which make the boundary became more difficult to define. However, after using the reduced POS tagging, the problem is solved and the accuracy of the “Operation” entities increases a lot, which reveals the effectiveness of our method.

We plot the length distribution of the correct results from SM-LSTM-CRF using each of the three types of input, shown in Table 8. It shows that the models involving part-of-speech tagging methods perform well in the entity length of “1–5” characters, and start to have an adverse effect when the entity length increases. After processed by the reduced POS tagging method, the part-of-speech information can simultaneously improve the recognition effects on both the long entities and the short entities, which are largely due to the boundary information of unknown clinical entities obtained through the known generic terms. It is justified to say that considering the currently poor states of medical dictionary construction and medical part-of-speech tagging, we turn to involve other information in the basic natural language processing to better mine the health information from the electronic medical records.

**Table 8:** The distribution of entities with various length in the correct results

	1–5	6–10	10–15	> 15
SM-LSTM-CRF(char)	6886	667	52	4
SM-LSTM-CRF(char + POS)	7440	485	21	3
SM-LSTM-CRF (char + Reduced POS tagging)	7335	673	59	3
Standard set	9014	1002	104	12

## Conclusions

This paper proposes a SM-LSMT-CRF model with reduced POS tagging for NER task on Chinese EMRs, which can handle the long-distance dependencies problem by implementing the Self-matching attention mechanism that helps to improve the accuracy of the long entity boundary recognition. With the reduced POS tagging method, the POS feature exhibits good performance for both short entities and long entities. Our empirical evaluation shows that our model can achieve a much better performance in Chinese EMRs than other algorithms.

## Abbreviations

CNN: Convolutional Neural Network
CRF: Conditional Random Field
EMR: Electronic medical record
LSTM: Long Short-Term Memory
NER: Named entity recognition
POS: Part Of Speech
